# Extra bone chondroma of the shoulder: a case study and literature review

**DOI:** 10.11604/pamj.2019.32.75.12804

**Published:** 2019-02-12

**Authors:** Soumaya Nasri, Narjisse Aichouni, Mounir Yahyaoui, Omar Agoumi, Imane Kamaoui, Imane Skiker

**Affiliations:** 1 Department of Radiology, CHU Mohamed VI, Oujda, Morocco; 2 Department of Traumatology, CHU Mohamed VI, Oujda, Morocco

**Keywords:** Soft tissue, extra-osseous chondroma, shoulder, MRI

## Abstract

Extra-osseous chondroma is a benign and rare tumor. It usually sits at the extremities, we report an exceptional case of a chondroma of the soft parts of the shoulder in a 28 year old woman who manifested by a painless swelling of the left shoulder. The histology confirmed the diagnosis on the excision piece. Clinical and radiological follow-up after a 24-month follow-up did not show a sign of recurrence.

## Introduction

Chondroma is a frequent benign tumor generally located at the bone level. Its extra-osseous location is extremely rare [1, 2]. The chondroma of the soft parts is mainly seated in the hand or the foot; its evolution is slow [2, 3]. It is formed of well defined cartilaginous nodules that develop in its soft parts, without adhesion to the bone or perioste which differentiates it from the juxta-cortical or periosteal chondroma. We report an exceptional case of extra-osseous chondroma of the shoulder by focusing on the clinical, radiological and histological signs and providing a literature review.

## Patient and observation

**Figure 1:**
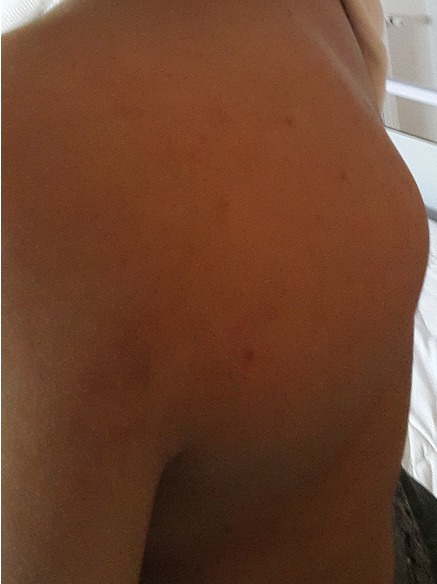
Tumefaction of the posterior aspect of the shoulder

**Figure 2:**
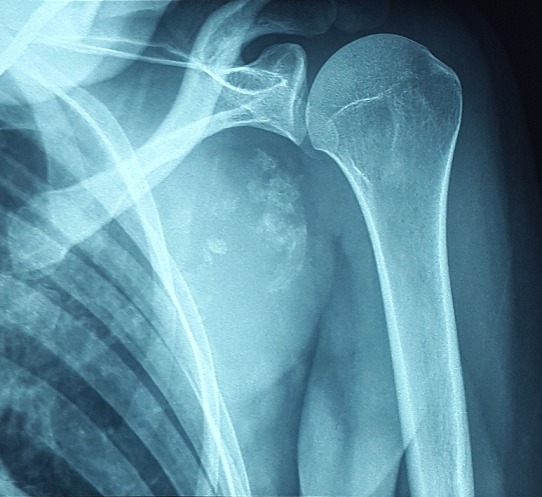
Standard radiograph of the shoulder showing the mass in calcium tonalite

**Figure 3:**
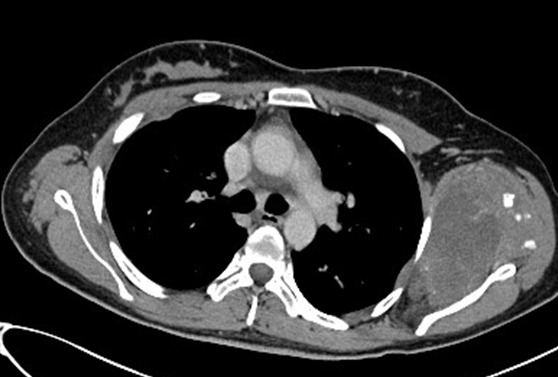
Mass at the dens of the left subscapularis muscle, polylobe heterogeneous hypodense containing calcification; thoracic TC in axial section after PDC injection

**Figure 4:**
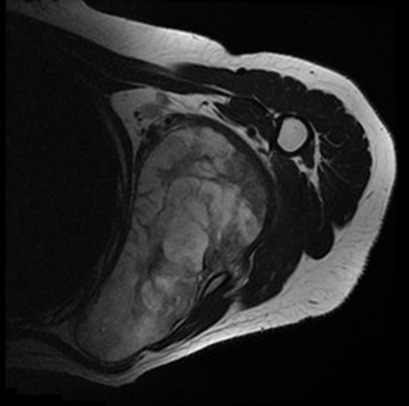
MRI of the shoulder lesional process of the sub-scapûlar fossa in hypersegnal T2 heterogenepus containing multible incompleten partitions enhanced after injection of the gadolinium leading necroses beacher, this process is limited by a pseudo-capsule: axial section T2

**Figure 5:**
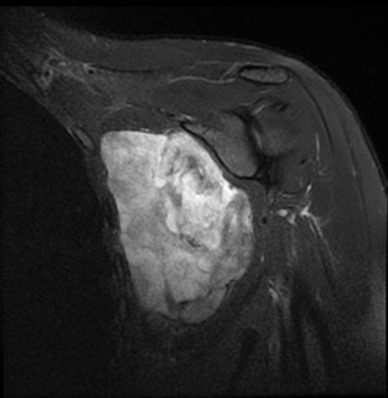
Coronal section DPFS

**Figure 6:**
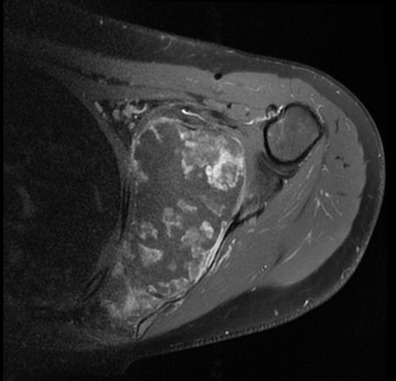
Axial section T1 gadolinium

**Figure 7:**
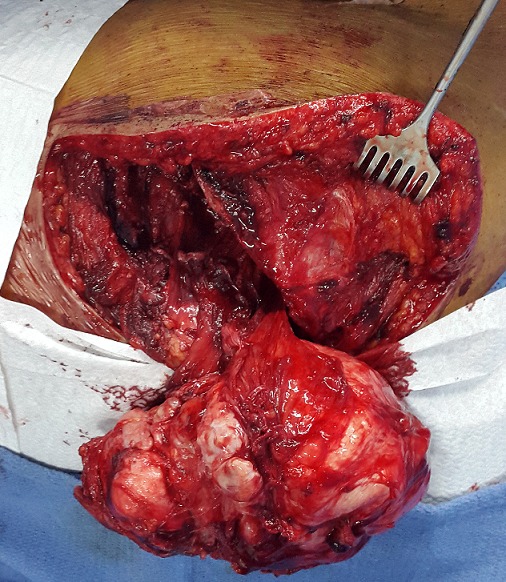
Peroperative image objectifying the tumor

A 28 year old female patient sought medical advice due to a painful tumefaction of the left shoulder which appeared four years ago and slowly increased in volume. The medical examination revealed that the patient did not have an old trauma nor did she have any medical or surgical antecedents. The physical examination showed a visible tumefaction (Figure 1), on the posterior side of the left shoulder. It is 20cm long; it is not well defined; and it is hard, painless, fixed compared to the two other plans. The mobility of the left shoulder is maintained but it is painful .The ganglionic areas are free. The somatic examination did not reveal any particularity. The chondroma evolves in a context of apyrexia and conservation of the general state. The radiography of the shoulder showed an image of calcium tone sitting in the soft parts without attachment to the periosteum (Figure 2). The CT scan individualized the presence of a lesional process of the left sub-scapular fossa; it is well-defined and it developed in the subscapularis muscle. It has a pharu-labile form; it is heterogeneous and hypodense and it contains multiple and sometimes incomplete partitions and calcifications. This process is increases heterogeneously after the injection of the contrast agent and the delimitation of the fluid areas (Figure 3). The MRI revealed a lesional process of the left sub-scapular fossa in isosignal T1, hypersignal T2, heterogeneous, containing multiple incomplete partitions heightened after the injection of gadolinium leaving necrosis spots and hypo-signal T2 foci that corresponded to calcifications. The process is limited by a pseudo-capsule and measures 13/09/10 cm (Figure 4, Figure 5, Figure 6). The surgical excision of the tumor was easy. In preoperative, the tumor was hard, well defined, poly-lobbed, and stuck to the front side of the scapula (Figure 7). The macroscopic examination reveals a mass measuring 12 x 10 x 10cm with slightly irregular contours and a crudely well circumscribed aspect in periphery. At the cut, there is a cartilaginous consistency and aspects in cauliflower of white, pearly color. The histological study of the various extractions shows a cartilaginous tumor proliferation organized in lobules. The cellularity within these lobules is moderate made up of discretely anisocaryotic chondrocytes, sometimes binucleated and whose periphery presents an enchondral ossification. The overgrowth is surrounded by a thick capsule. After a 24 months follow up, the patient is asymptomatic without any sign of recurrence at the MRI examination.

## Discussion

Extra-osseous chondromas are relatively rare. They may occur at any age but are predominantly frequent between the third and seventh decades [4, 5], generally located in contact with the peri-articular tissues or tendon sheaths without adhesion to the bone, almost always in the extremities and often in the hands [6]. No case of extra-osseous chondroma of the shoulder has been reported in the literature. The majority of publications on extra-osseous chondroma describe isolated observations or small series. Two studies of 70 and 104 patients [6] respectively confirm the benign nature of this tumor, its predilection for the distal part of the extremities and the variable character of the histological aspect which is often wrongly imposed on a Chondrosarcoma [7]. The etiology of these tumors remains in most cases not determined, it is thought to be a proliferation of the synovium and some authors relate it to the synovial chondromatosis, which generally affects the large joints [8]. The tumor is unique in the vast majority of cases, although some bilateral forms have been described [9]. In case of multiple lesions, it is often in fact, a synovial chondromatosis. The CARNEY Triad associates a pulmonary chondroma, a gastric leiomyosarcoma and, an extra-surrenial paraganglioma [10]. On the clinical level, it is a painless swelling of the soft parts slowly increasing in volume so that the patient does not immediately seeks medical advice [11, 12]. On the radiological level, the appearance of the extra-osseous chondroma varies according to the size of the calcification of the tumor center and the reaction of the adjacent tissues. Calcification usually develops in about a one-third of the cases in the centre of the lesion. Bone lesions are rare although the mass can be responsible for erosions and cortical changer [13, 14].

At the MRI, peripheral contrast is traditionally observed in the extra-osseous chondroma, but adjacent soft tissues are normal. A homogeneous signal mainly hypertensive on the T2 sequences is observed in most tumors. It is therefore a non specific sign that is not particularly favorable for a benign cartilage mass. On the histological level, the macroscopic examination usually shows a lobulated well-encapsulated rubbery tumor, which is sometimes more friable with focal cystitis. The size is small and rarely superior to 3cm. While microscopic examination shows the presence of chondrocytes which are marked by the anti-protein antibody S100 [15], electron microscopic examination shows chondrocytes containing large dental nuclei, an abundant rough endoplasmic reticulum, and in some cases vacuoles attached to the membrane. Short microvilli or filopodia spread from the cell membrane into the neighboring fundamental substance. The latter contains in the case of calcified tumors, aggregates of crystals of hydroxyapatite of variable size. We rarely observe myxoid rearrangements, zones of increased cellulariy and large chondrocytes causing fear of a malignant lesion [15]. The variability of the histological aspect may incorrectly refer to an extra-osseous chondrosacrome, especially in the case of myxoid rearangements or more rarely a synovial sarcoma of the soft parts. The synovial sarcoma is the soft-tissue sarcoma, which is most often confused with extra-osseous chondroma. It is also appropriate to eliminate some benign lesions such as tumor calcinosis, a synovial chondromatosis, a gouty tophus, a lipoma containing areas of metaplasia chondroid, a pilomatricoma, an ossifying myositis an advanced stage, or a soft-tissue mass related to a melorheostose. The treatment of this tumor is the surgical excision without biopsy beforehand considering the criteria of benignity and the easy resection of the tumor [16]. No malignant transformation of an extra-osseous chondroma has been described before. Despite the benign character of the lesion, a local recidivism occurs in 15 to 25% of the cases [17] because of an incomplete excision or because of uncertainty with regard to the histological nature. A new excision is the best solution in the case of tumor recurrence.

## Conclusion

The chondroma of the soft parts is a rare lesion; its localization at the level of the shoulder is exceptional. The diagnosis is made at the clinical and radiological examinations. A complete excision of the tumor constitutes the treatment of choice and recurrences are exceptional.

## Competing interests

The authors declare no competing interests.
